# Analysis of quality of life and outcomes of vestibular schwannoma patients after resection and radiosurgery in an interdisciplinary treatment concept

**DOI:** 10.1038/s41598-025-00372-0

**Published:** 2025-05-07

**Authors:** Alexander Romagna, Christoph Schwartz, Beate Huffmann, Wolfgang Hitzl, Rudolf A. Kristof, Hans Clusmann, Christian Blume

**Affiliations:** 1 Department of Neurosurgery, München Klinik Bogenhausen, Munich, Germany; 2 Department of Neurosurgery, University Hospital Salzburg, Paracelsus Medical University, Salzburg, Austria; 3 Department of Neurosurgery, RWTH Aachen University, Aachen, Germany; 4 Research and Innovation Management, Biostatistics, Paracelsus Medical University, Salzburg, Austria; 5 Department of Ophthalmology and Optometry, Paracelsus Medical University, Salzburg, Austria; 6 Research Program Experimental Ophthalmology and Glaucoma Research, Paracelsus Medical University, Salzburg, Austria; 7 Department of Neurosurgery, HELIOS Hospital Meiningen, Meiningen, Germany

**Keywords:** Interdisciplinary treatment concept, Gamma knife radiosurgery, Microsurgery, Vestibular schwannomas, Quality of life, CNS cancer, Outcomes research

## Abstract

Quality of life (QoL) is a crucial factor which has to be taken into account in the treatment of vestibular schwannomas. This study compared microsurgical and radiosurgical treatments, focusing on three. (1) evaluating post-treatment clinical outcomes, (2) assessing the effect on QoL and (3) analyzing complication rates, particularly in geriatric patients. In this retrospective study, 586 patients underwent either microsurgery or gamma knife radiosurgery between 1990 and 2013. Demographic and treatment data were collected, including quality of life (QoL) assessments using the Short Form 36 (SF-36) and evaluations of the Karnofsky Performance Status (KPS). Complication rates were also analyzed. The study included 194 microsurgery patients and 392 radiosurgery patients. Radiosurgery patients showed significantly better postoperative hearing and facial nerve function (*p* < 0.05). QoL scores were higher in the radiosurgery group for physical and emotional role functioning, while microsurgery patients (including aged 65 years and older) reported better scores for bodily pain and general health perceptions. Neurological complications were 41.9% in the microsurgery group, most of them being permanent (92.9%). Radiosurgery generally showed better preservation of hearing, facial nerve function, and quality of life compared to microsurgery, although both treatments appeared equally effective in older patients.

## Introduction

Minimizing treatment burden and preserving the patients’ quality of life (QoL) is, besides tumor control, paramount in vestibular schwannoma management^1^. While treatment options to achieve complete tumor removal or long-term tumor control have evolved, the treatment priority remains unchanged: avoiding complications and safeguarding patients’ QoL^2^. Current strategies to achieve these goals are multidisciplinary and personalized. Microsurgery by a suboccipital-retrosigmoid approach utilizing intraoperative neurophysiological monitoring (IOM)-guidance as well as gamma knife radiosurgery (GKRS) are well established treatment standards. Both, however carry relevant treatment-associated risks^3,4^. In this context, recent literature suggests that microsurgery may initially negatively impact QoL with subsequent improvement seen over time, while GKRS has a less negative effect with minimal change during follow-up on patients’ QoL^5^. Still, there is no consensus on which aspects of QoL are most affected or the role of factors such as tumor size, cranial nerve function and age^6^. With regards to the latter, evidence shows that the geriatric subpopulation (> 65 years) is particularly vulnerable to treatment-associated complications since pre-existing comorbidities and patient frailty complicate treatment and recovery^7^. However, vestibular schwannomas are known to cause significant disabilities in the elderly when left untreated^7,8^. In this context, the SF-36 has been the most widely used comprehensive measure of QoL and a valuable tool for comparing outcomes across different treatment modalities throughout different age groups^9^. By retrospectively analyzing data spanning over two decades, this study aimed to compare the effects of microsurgical and GKRS treatment of vestibular schwannomas on QoL and treatment-associated complication rates.

## Methods

### Patients and outcome analysis

This was a retrospective study conducted at a single interdisciplinary center. Following approval from the institutional review board (EK 125/16) the tumor registry was queried from February 1990 to November 2013 for patients who underwent either microsurgery or GKRS for unilateral vestibular schwannoma at an academic interdisciplinary center. Patients who were previously treated with radiotherapy were excluded from this analysis. Clinical outcome analysis focused on caudal cranial nerve (CN) function (i.e. CN V, CN VI, CN VII, CN VIII and CN IX impairment), the incidence of hydrocephalus/shunt dependency and gait ataxia. Facial nerve function was assessed by the House-Brackmann Facial Nerve Grading System: grades I-III were considered good facial function^10^. Hearing function was differentiated into normal hearing, hypacusis, and normal hearing as assessed by pure tone and speech audiometry^11^. Furthermore, the presence of patient reported tinnitus was recorded. The patients’ QoL analysis was performed by the Short Form Health Survey (SF-36). Additionally, the patients’ functional status was assessed by the Karnofsky Performance Status (KPS) and treatment-associated complications were recorded.

### Treatment

The treatment modality for all patients was recommended on a case-by-case basis by an interdisciplinary neurooncological tumor board. All patients who underwent microsurgery were operated using the retrosigmoid-suboccipital approach as previously described and IOM was routinely used in all procedures^12^. In brief, GKRS was performed using a Leksell Gamma Knife model B (Elekta Instrument AB, Stockholm, Sweden)^13^. Multishot-dose plans were created to construct a radiation field conformal with the tumor shape. Treatment planning was performed using Leksell Gamma Plan (Elekta Instrument AB) as described previously^14^. The median applied dose was 12.7 Gy (range 11.0–14.0 Gy).

### Data collection

**Fig. 1:**
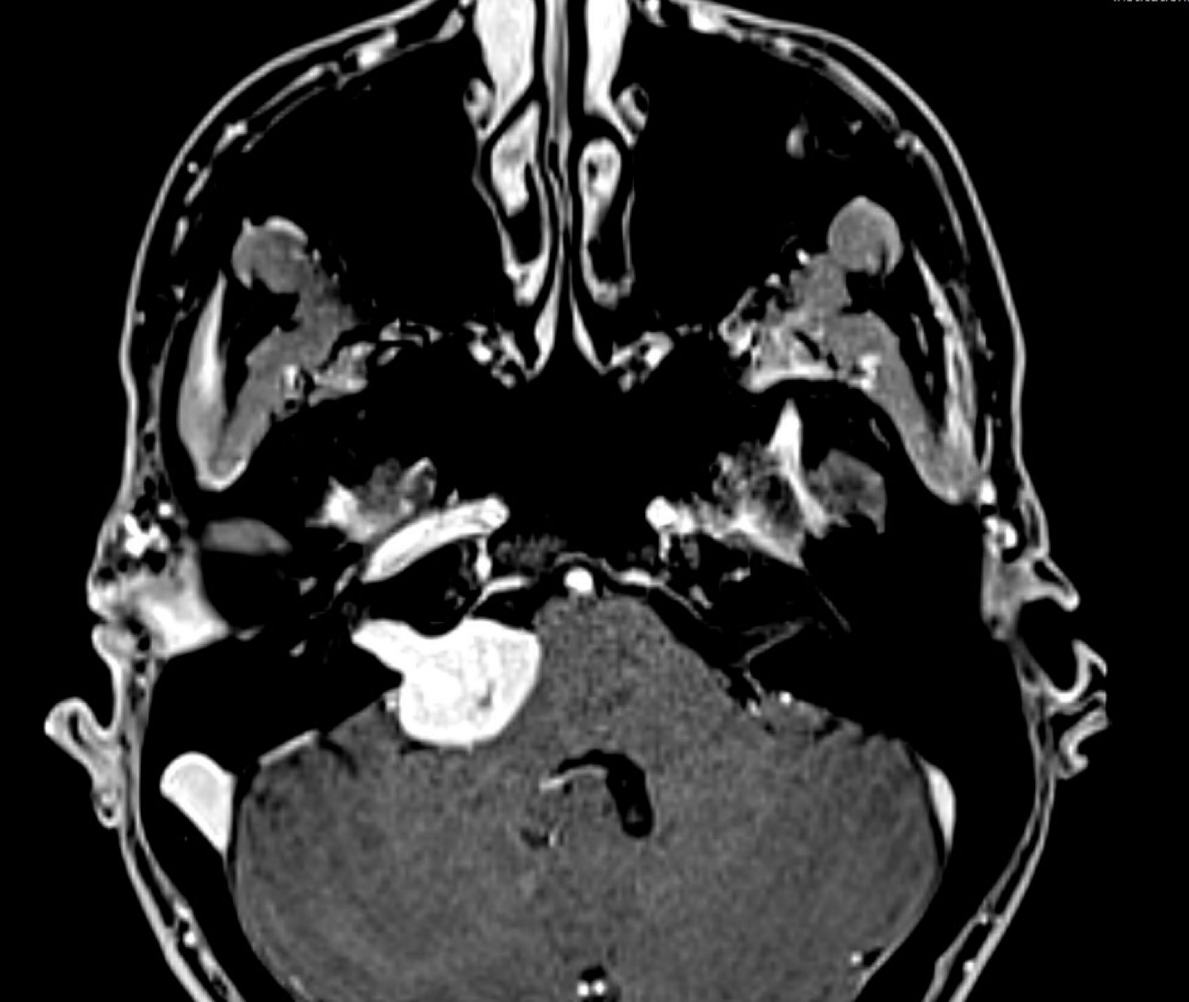
Illustrative case. T1-weighted MR imaging of a 70-year old female patient diagnosed with a right sided vestibular schwannoma.

Tumor size was measured radiographically using contrast-enhanced magnetic resonance (MR) imaging (see illustrative case in Fig. 1). At least two senior neuroradiologists independently evaluated the tumor sizes in a blinded manner preoperatively, 6 months post-treatment and at the final follow-up. Clinical-neurological follow-up assessments were conducted at discharge and 3 to 6 months post-treatment, including evaluations of CN function as well as assessments for hydrocephalus and ataxia. Separate analyses were performed for elderly patients aged 65 years and older. SF36 evaluation was performed 9–12 months after treatment (median 10.5 months).

### Statistical analysis

Data were checked for consistency and normality using Saphir-Wilks test. Fisher’s Exact test or Pearson’s Chi Squared test were used to analyze cross tabulations. In case of normal distributions, independent t-tests were used, otherwise bootstrap-t tests to compare means. Marginal homogeneity test was used to test the change of tumor sizes (smaller, stable, larger) over time. Linear and logistic regression analyses were also applied and illustrated using Scatterplots. All reported tests were two-sided, and p-values < 0.05 were considered statistically significant. All statistical analyses in this report were performed by use of STATISTICA 13 (Hill, T. & Lewicki, P. Statistics: Methods and Applications. StatSoft, Tulsa, OK) and PASW 27 (IBM SPSS Statistics for Windows, Version 27.0., Armonk, NY). Data analysis was performed as per protocol and all statistical analyses were performed by a biostatistician (WH).

## Results

### Patients

The total study population consisted of 586 patients (308 females) with a median age at treatment of 57.5 years (IQR 47–68 years) and a mean KPS of 89.6 ± 5.8 prior to treatment. The microsurgery cohort consisted of 194 (33.1%) cases and 392 (66.9%) patients were treated by GKRS. Among patients who underwent GKRS were 17.6% cases who had undergone prior surgery and in accordance with the inclusion criteria no patients had received prior radiotherapy. The median pre-treatment KPS was 90.2 ± 9 and 89.3 ± 3.1 respectively, for the microsurgery and GKRS groups (*p* = 0.18). Patients who underwent microsurgery had larger tumors (2.6 vs. 1.8 cm^3^; *p* < 0.0001) and more often of female sex (59.8 vs. 49%; *p* = 0.017). Furthermore, patients aged 65 years and older were more common (24.2 vs. 36.4%; *p* = 0.03) among the GKRS cohort (data summarized in Table 1).

**Table 1:** Basic population characteristics.

	All patients *n* = 586	Microsurgery *n* = 194	Radiosurgery *n* = 392
Median age (years) (IQR)	57.5 (47–68)	52.5 (43–64)	59 (48–69)
Age ≥ 65 yrs	489 (32.2%)	47 (24.2%)	142 (36.4%)
Sex			
Male	278 (47.4%)	78 (40.2%)	200 (51%)
Female	308 (52.6%)	116 (59.8)	192 (49%)
Tumor size (cm^3^)	2 (1.5–2.6)	2.6 (2–3.5)	1.8 (1.4–2.3)
Median prescription dose Gy	12.7 (0.90)	–	12.7 (0.90)

No significant differences between treatment groups for all evaluated parameters were detected (*p* > 0.05), beside sex (*p* = 0.017) and tumor size (*p* < 0.0001) and age ≥ 65 yrs (0.03)

With regard to CN functions the following status were recorded prior to treatment: the share of patients with hypacusis and anacusis were 62.9% and 33% in the microsurgery cohort and the respective rates were 74.1% and 22.8% in the GKRS cohort. Thus, pre-treatment anacusis was significantly more common in patients treated by surgery (*p* = 0.009). Normal hearing function and tinnitus prior to treatment were equally distributed (*p* > 0.05). Good facial nerve function was seen in 193 (99.5%) patients of the microsurgery cohort and 376 (96.4%) patients of the GKRS cohort (*p* = 0.016; Tables 2 and 3). No pre-treatment differences in CN V, CN VI and CN IX function as well as incidence of hydrocephalus and ataxia between cohorts were detected (all *p* > 0.05) In the subgroup of patients > 65years, hydrocephalus was more frequent in the microsurgery group preoperatively (17% vs. 4.9%; *p* = 0.01), but not postoperatively (3% vs. 8%; *p* = 0.43). The same was seen for ataxia both before and after treatment overall.

**Table 2:** Hearing function outcome parameters.

Preoperative clinical status*	All patients *n* = 583	Microsurgery *n* = 194	Radiosurgery *n* = 390	*p*-value between treatment groups
Anacusis	153	64 (33%)	89 (22.8%)	0.009
Hypacusis	411	122 (62.9%)	289 (74.1%)	0.0069

Clinical Status at last follow up*	All patients *n* = 420	Microsurgery *n* = 96	Radiosurgery *n* = 324	*p*-value between treatment groups
Anacusis	247	24 (25%)	223 (68.8%)	*p* < 0.0001
Hypacusis	10	3 (3.1%)	7 (2.1%)	1.0

*Data correspond to the available dataset for patients at preoperative and last follow-up assessments

**Table 3:** Facial function outcome parameters.

	All patients *n* = 586	Microsurgery *n* = 194	Radiosurgery *n* = 392	*p*-value between treatment groups
Preoperative clinical status*				
Good facial function	569 (97.4%)	193 (99.5%)	376 (96.4%)	0.016
Clinical Status at last follow up*				
Good facial function	388 (92.22%)	76 (78.4%)	312 (96.3%)	*p* = 0.0001

*Data correspond to the available dataset for patients at preoperative and last follow-up assessments

### Functional impairment and QoL

Performance status measured by the KPS remained consistent across groups both before and after treatment, showing virtually no change. Neither being 65 years or older nor having a higher tumor volume were prognostic factors for low KPS throughout the follow-up period (*p* = 0.84 and *p* = 0.73 for all groups). Quality of life data, measured using the SF-36, revealed that patients in the microsurgery group reported higher QoL scores at the last follow-up (median 10.5 years) for “bodily pain” and “general health perceptions” in comparison to the gamma knife radiosurgery group, both overall and in patients aged 65 years and older. The radiosurgery group showed overall better scores for “physical functioning,” “physical role functioning,” and “emotional role functioning” (data summarized in Table 4). Neurological complications were 41.9% in the microsurgery group. Most of them were permanent (92.9%). In the radiosurgery group, the proportion of those whose tumors decreased in size increased from 15.4% (6 months) to 28.9% (last follow-up; *p* < 0.00001).

**Table 4:** Longitudinal clinical outcome and quality of life parameters.

	All patients *n* = 586	Microsurgery *n* = 194	Radiosurgery *n* = 392	*p*-value between groups
Karnofsky performance status				
Prior to treatment	89.6 (5.8)	90.2 (9)	89.3 (3.1)	0.18
3 months after treatment	89.3 (10.2)	89.1 (11.1)	90 (0)	1.0
At last follow up	88.7 (7.7)	89.6 (7.1)	88.4 (7.9)	0.20
SF-36 after treatment				
Vitality	53.0 (12.7)	53.6 (10.6)	51.5 (11.7)	0.20
Physical functioning	77.5 (25)	70.3 (26.8)	82.1 (22.7)	0.001*
Bodily pain	19.9 (24.2)	24.7 (26.1)	16.3 (21.3)	0.014*
General health perceptions	55.8 (13.7)	59.9 (12.9)	53.2 (13.7)	0.001*
Physical role functioning	65.5 (40.4)	56.2 (41.6)	71.2 (38.6)	0.01*
Emotional role functioning	76.1 (39.2)	68.9 (43.7)	80.4 (35.8)	0.044*
Social role functioning	46.8 (10.4)	44.8 (10.4)	47.7 (9.2)	0.03*
Mental health or emotional wellbeing	61.9 (10.9)	60.6 (12.4)	62.6 (9.7)	0.12
Time of SF-36 survey after treatment (months)	10.5 (5.0)	10.2 (6.9)	10.7 (3.4)	0.52

Numbers in parentheses indicate standard deviations.

## Discussion

### Key findings

Vestibular schwannomas require careful treatment planning to balance tumor control with preservation of neurological function and QoL^15^. Our study focused on comparing outcomes between microsurgery and radiosurgery, revealing several key findings: (1) GKRS demonstrated superior postoperative hearing and facial nerve preservation, (2) QoL scores, particularly in physical and emotional role functioning, were higher in the radiosurgery group, and (3) neurological complications were more common and often permanent in the microsurgery cohort.

### Clinical outcome

Regarding hearing and facial nerve function, our results align with existing literature that underscores the advantages of radiosurgery and reports about its CN VII preservation rates^16,17^. However, when reviewing the data from the geriatric subgroup only (65 years and older), no significant differences in postoperative outcomes for facial nerve function, hearing preservation, and complications related to CN VI and CN IX impairments between the two treatment modalities could be seen. This is in line with previous studies suggesting that older age does not necessarily compromise the surgical outcomes of vestibular schwannoma treatments^18,19^. The influence of age on outcomes is multifaceted and might be affected by co-morbidities in older patients or compensatory mechanisms in younger ones. The timing of postoperative assessments may also play a role, as later assessments may reflect complications or age-related frailty, making direct comparisons difficult.

Our analysis of QoL, as assessed by SF-36, revealed notable distinctions between the two treatment modalities. Patients undergoing GKRS consistently reported higher QoL scores in physical and emotional role functioning compared to those treated with microsurgery. This might suggest that radiosurgery may offer patients better functional outcomes, i.e. maintaining daily activities and emotional well-being post-treatment. Conversely, microsurgery patients reported higher QoL scores in domains related to bodily pain and general health perceptions. This might imply that microsurgery could offer advantages in terms of pain management and overall health perceptions. Of note, patients aged 65 years and older who underwent microsurgery reported higher QoL scores in domains related to bodily pain and general health perceptions as well. This might indicate that despite potentially higher complication rates, the geriatric subpopulation may experience less discomfort and have a better overall perception of their health status post-treatment compared to their counterparts who underwent radiosurgery. Ultimately, it is possible that factors such as dizziness, tinnitus and facial nerve deterioration play a significant role in impacting quality of life, particularly in physical and emotional functioning. Additionally, hearing loss could further contribute to these challenges, but these hypotheses warrant further investigation.

### Limitations

This study is limited by its retrospective design and the long timeframe (1990–2013). Consequently, advancements in surgical and radiosurgical techniques may influence current outcomes, limiting the applicability to present-day practices^2^. Moreover, SF-36 data were only collected postoperatively and not preoperatively for all patients, which limits the ability to assess changes in quality of life attributable to the treatment itself. Nevertheless, our data emphasize the significance of tailored treatment decisions, particularly for older patients suffering from vestibular schwannomas.

## Conclusions

This study underscores the benefits of GKRS in preserving hearing, facial nerve function, and certain quality of life aspects, while microsurgery excels in pain management and general health perceptions. Future studies incorporating recent data are needed to reflect advancements and ultimately improve patient outcomes.

## Data Availability

All data that support the findings of this study are available from the corresponding author upon reasonable request.
